# Endoplasmic Reticulum Is at the Crossroads of Autophagy, Inflammation, and Apoptosis Signaling Pathways and Participates in the Pathogenesis of Diabetes Mellitus

**DOI:** 10.1155/2013/193461

**Published:** 2013-05-21

**Authors:** Jing Su, Lei Zhou, Xiaoxia Kong, Xiaochun Yang, Xiyan Xiang, Yu Zhang, Xiaoning Li, Liankun Sun

**Affiliations:** ^1^Department of Pathophysiology, Norman Bethune College of Medicine, Jilin University, Changchun 130021, China; ^2^Department of Pathology, Affiliated Hospital to Changchun University of Chinese Medicine, Changchun 130021, China; ^3^Institute of Hypoxia Research, School of Basic Medical Sciences, Wenzhou Medical College, Wenzhou, Zhejiang 325035, China

## Abstract

Diabetes mellitus (DM) is a chronic metabolic disease, and its incidence is growing worldwide. The endoplasmic reticulum (ER) is a central component of cellular functions and is involved in protein folding and trafficking, lipid synthesis, and maintenance of calcium homeostasis. The ER is also a sensor of both intra- and extracellular stress and thus participates in monitoring and maintaining cellular homeostasis. Therefore, the ER is one site of interaction between environmental signals and a cell's biological function. The ER is tightly linked to autophagy, inflammation, and apoptosis, and recent evidence suggests that these processes are related to the pathogenesis of DM and its complications. Thus, the ER has been considered an intersection integrating multiple stress responses and playing an important role in metabolism-related diseases including DM. Here, we review the relationship between the ER and autophagy, inflammation, and apoptosis in DM to better understand the molecular mechanisms of this disease.

## 1. Introduction

Diabetes mellitus (DM) is a chronic metabolic disease, and its incidence is growing worldwide. Long-term hyperglycemia is the fundamental factor that promotes vascular lesions and dysfunction, leading to a variety of complications of DM [1]. Diabetic complications, such as neuropathy vasculopathy, are the main cause of death or disablement in DM patients [2]. The main purpose of clinical treatments for DM is to control blood glucose and consequently inhibit or alleviate the initiation and progression of complications. However, the control of blood glucose is not easy to achieve [3]. Therefore, a better understanding of the pathogenesis of DM is very important for the development of new treatment strategies. 

The endoplasmic reticulum (ER) is an important membranous organelle; its functions include folding and trafficking of protein, lipid synthesis, maintaining calcium homeostasis, and participating in a number of crucial cellular functions [4]. The ER can monitor and maintain cellular homeostasis by acting as a sensor of various changes (stresses) in the intra- and extracellular environment [5]. The ER may therefore provide a platform for interactions between environmental signals and basic cellular biological functions and act as an intersection to integrate multiple stress responses. The interruption of cellular homeostasis can lead to a gradual reduction of organ function, and in turn decreased ability to respond to physiological stress. Recently, a growing body of research has suggested that the ER is involved in the pathogenesis of DM and its complications [6, 7]. Additional research is required to investigate the roles of the ER and its related signaling networks in DM and to thus help develop novel therapeutic strategies. 

## 2. The Unfolded Protein Response and ER Stress

The ER is an important center of multiple cellular processes; it has the ability to regulate synthetic, metabolic, and adaptive responses to both intra- and extracellular stress and plays a crucial role in maintaining cell homeostasis. When unfolded or misfolded proteins accumulated in the ER lumina, an adaptive response called the unfolded protein response (UPR) occurs [8]. The typical UPR consists of three pathways in eukaryotic cells, which are mediated by three ER membrane-associated proteins: PKR-like eukaryotic initiation factor 2a kinase (PERK), inositol requiring enzyme 1 (IRE1), and activating transcription factor-6 (ATF6). These sensors can monitor changes in the ER lumen and activate downstream signaling pathways. Under stress-free conditions, these sensors are combined with the ER chaperone Bip/GRP78 (glucose regulated protein 78) and exist in their deactivated form [9, 10]. When misfolded proteins accumulate in the ER lumina, UPR sensors detach from GRP78, causing oligomerization and activation of PERK and IRE1 and leading to the activation of downstream signaling pathways [8]. ATF6 is translocated to the Golgi apparatus, where processing by serine protease site-1 protease (S1P) and serine protease site-2 protease (S2P) produces a new active transcription factor [11]. Under ER stress, ATF6 is reduced, and only reduced ATF6 can translocate to the Golgi apparatus, indicating that redox state is one of the factors that determines activation of ATF6 [12]. The UPR can alleviate ER stress by reducing protein synthesis, promoting protein degradation and producing chaperones to assist with protein folding [13]. Excessive or prolonged ER stress can lead to cell death mediated by apoptosis [14].

To date, studies investigating the roles of UPR and ER stress in human diseases have mainly focused on the PERK and IRE1*α* pathways. Because of the lack of effective research methods and pharmacological tools, the available data regarding the potential role of ATF6 are not sufficient. The adaptability of ER dysfunction can cause UPR activation, and the UPR and ER stress are linked to many different stress signaling pathways [15–17]. This indicates that the ER may be an intersection at which the integration of multiple stress reactions occurs, and it may play an important role in the pathogenesis of chronic metabolic diseases such as type 2 diabetes. 

## 3. ER Stress and Autophagy

Autophagy is a highly conserved and tightly regulated cellular process. Autophagy is a pathway that allows energy/constituent recycling. It also participates in the degradation of misfolded proteins and damaged organelles and facilitates cellular health under various stress conditions including hypoxia, ER stress, or oxidative stress [18–20]. Although the role of autophagy in normal ER function is not established, there are some studies that have shown that autophagy is associated with the ER and maybe an important part of normal ER function [21, 22]. ER stress-induced autophagy plays an important role in maintaining cellular homeostasis through alleviating stress. The ER is related to two major degradation processes in eukaryotic cells: the ubiquitin-proteasome pathway and the autophagy-lysosome pathway [23]. ER stress-induced autophagy can also be used as an alternative degradation mechanism to process misfolded proteins that have accumulated in the ER lumen and that could not be removed by the endoplasmic-reticulum-associated protein degradation (ERAD) pathway. Under over nutrient, high mTOR activity suppresses the mammalian autophagy-initiating kinase Ulk1 by phosphorylating Ulk1 Ser 757 and disrupting the interaction between Ulk1 and AMPK. It suggests that obesity can regulate autophagy through the mTOR signaling pathway [24]. However, under conditions of excessive nutrition, the regulatory mechanisms of autophagy and its effects on insulin action still need further study. 

Autophagy plays important roles in metabolic organs, and abnormal autophagy is involved in the pathogenesis of metabolism-related diseases including diabetes and kidney disease [25]. It has been shown that autophagy is essential to islet function and survival and that autophagy deficiency can lead to islet degeneration and reduced insulin secretion [26, 27]. Sequestosome 1 (SQSTM 1/p62) is an important autophagy-related protein. SQSTM 1/p62 deficient mice exhibit metabolic abnormalities and diabetes [28, 29]. Suppression of autophagy can lead to accumulation of reactive oxygen species in the mitochondria, and this may cause initiation of early diabetic nephropathy. Autophagy deficiency in kidneys of diabetic animals can lead to tubule cells being vulnerable to hypoxia and ER stress and can result in progression of diabetic nephropathy [25]. Activation of autophagy may therefore be one therapeutic option for end-stage diabetic nephropathy. Autophagy plays a major role in fatty liver which is directly involved in type II diabetes complications. Autophagy is insufficient in hepatocytes in obesity or fatty liver patients. Its upregulation improves insulin sensitivity and maybe helpful in the treatment of DM [30].

## 4. ER Stress and Inflammation

Inflammation is involved in the mechanism of metabolism-related diseases such as DM and obesity [31, 32]. Controlling the inflammatory process can therefore be a potential therapeutic target to inhibit the progress of metabolism-related diseases. ER stress and UPR signaling pathways are linked with some of the major inflammation and stress signaling networks including the JNK-AP1 and NF-*κ*B-IKK pathways [33, 34]. In JNK-deficient mice, the expression of proinflammatory cytokines (including TNF*α*, IL-6, and MCP-1) induced by obesity is suppressed, and this can promote protection from insulin resistance and T2DM (type 2 diabetes mellitus) [35–38]. The IRE1*α* and PERK pathways of UPR can activate the NF-*κ*B-IKK signaling pathway (inducing the expression of a wide variety of inflammatory mediators) and participate in insulin resistance [39]. Recently, it was shown that the ATF6 pathway is also linked with the NF-*κ*B-IKK pathway, and it has been suggested that specific inflammatory activators can transmit signals through different UPR pathways [40]. 

The relationship between ER stress and inflammation is not one-way. The activation of inflammatory mediators and cellular stress signaling pathways, such as the JNK and IKK pathways, may have a negative impact on ER function [15, 41]. Notably, exposure to inflammatory cytokines such as TNF-*α* can induce ER stress, and ER stress itself can cause an increase in the expression of TNF-*α* or perhaps more general inflammatory responses [42]. However, the interactions between inflammation and ER stress may be different depending on cell type. Additional research is therefore needed to determine the sensitivity of ER homeostasis to inflammatory signals and the effects of inflammatory stress on ER homeostasis in different cells. 

The tight relationship between ER stress and inflammation integrates ER function and metabolic homeostasis and plays a crucial role in obesity, insulin resistance, T2DM, and other metabolic abnormalities [42]. Chronic inflammation can cause ER stress in adipose tissue, and trigger cell damage or death to some degree [3, 43–45]. In metabolic organs, how the ER and its integrated stress signaling system associate with the function of immune cells is unclear [46]. In chronic inflammation, there is evidence that metabolic cells (such as adipocytes and liver cells) can be driven by metabolic hormones and that this can interrupt immune effectors and diffuse inflammatory signaling [47]. However, studies are still needed to clarify the important roles of ER stress and inflammation in metabolism.

## 5. ER Stress and Apoptosis

If excessive and prolonged ER stress cannot be alleviated by the UPR, functional homeostasis of the ER cannot be reestablished, and this will induce cell death by apoptosis [14]. The PERK pathway activation can induce expression of the proapoptotic transcription factor *chop/gadd153* and activate caspase-12 and JNK signaling pathways, thus leading to apoptosis [48]. IRE1*α* pathway activation can initiate the proapoptotic Bcl-2 protein and activate Bax and Bak and can also cause tumor necrosis factor receptor-associated factor 2 (TRAF2) and apoptosis signal-regulating kinase 1 (ASK1) recruitment to the cytoplasmic side of the ER membrane, thus leading to apoptotic signaling [49, 50]. 

As mentioned above, PERK and IRE1*α* pathway activation can also regulate the NF-*κ*B-IKK signaling pathway by IKK activation or p65 degradation during ER stress. The ATF6 pathway can regulate activity of NF-*κ*B. All of these signaling pathways can trigger apoptosis during excessive or prolonged ER stress. Differences between activation of the three UPR pathways are likely to determine apoptosis [8]. For example, continuous ER stress can reduce IRE1 and ATF6 activity and activate PERK, leading to apoptosis [51]. Similarly, reduction of IRE1 RNA ribonuclease activity can suppress its protective role through producing XBP1 and trigger JNK activation or release proapoptotic mediators [52]. Degradation of mRNA located in the ER by IRE1*α* may be an important mechanism of ER stress that determines cell death or cell survival [53]. This indicates that IRE1 may be the key “lever” to determine cell fate. The roles of each UPR initiation factor in recognition and response to various types of ER stress are not completely understood. There is also a need for further research to establish how the different UPR pathways function under particular conditions and different cellular environments and to determine whether they participate in different reactions and produce different effects on cells. Although the major function of the ER is protein processing, factors in addition to protein overload can trigger UPR; interruption of ER calcium homeostasis, pathogens or pathogen-related components, or lipids and toxins can also induce ER stress and lead to apoptosis.

Apoptosis is involved in the pathogenesis and progression of DM, and pancreatic beta-cell apoptosis can cause islet degeneration and decrease insulin secretion. In human and experimental kidney diseases such as diabetic nephropathy, hypertensive nephrosclerosis, and glomerulonephritis, glomerular cells including mesangial cells and podocytes can upregulate apoptosis. Prevention of apoptosis may therefore help relieve the progress of DM and its complications.

## 6. ER Stress in DM

Lipid or inflammatory pathways can trigger insulin resistance, lead to abnormal glucose utilization and blood glucose levels, and promote ER stress. Recent studies have shown that ER stress is involved in obesity, insulin sensitivity, and DM and that the UPR plays an important role in the pathogenesis of these diseases. XBP1-deficient cells or mice are more sensitive to ER stress and insulin resistance because of PERK- and IRE1*α*-dependent JNK activation. This indicates that there is a causal relationship between sensitivity to ER stress and insulin resistance [54]. In contrast, cells with a higher level of XBP1 tolerate ER stress tolerance and show protective effects against insulin resistance[54]. The ER chaperone oxygen-regulated protein 150 (ORP150), which is activated by the UPR, plays a protective effect on maintaining metabolic homeostasis in mice [55, 56]. ORP150 deficiency in the whole body, particularly the liver, can impair glucose tolerance or reduce insulin receptor signaling by IRS1 phosphorylation [57]. In contrast, in obese or diabetic mice, ORP150 overexpression can lead to increased glucose tolerance and enhanced insulin receptor signaling. Similarly, GRP78 overexpression has beneficial metabolic effects, including reducing liver steatosis and increasing insulin sensitivity in the liver in obese mice [58]. In addition, EIF2*α* mutant heterozygote mice exhibit ER dysfunction, which leads to obesity and T2DM when fed a high-fat diet [59]. In short, studies based on these animals model provide evidence that ER function is linked to DM. 

The effects of ER stress and the UPR on pancreatic islet survival and function play crucial roles in the pancreas. For example, PERK-deficient pancreatic beta cells are more sensitive to apoptosis induced by ER stress. PERK-deficient mice exhibit severe hyperglycemia soon after birth because of a defect in pancreatic proliferation and increased apoptosis [60]. In pancreatic beta cells, suppression of eIF2*α* phosphorylation can also prompt DM progression which is supported to be secondary to oxidative stress [61]. ER chaperone p58 (IPK) deficiency can promote beta cell dysfunction [62]. In humans, the PERK-eIF2*α* pathway is very important to islet survival and function. In fact, Wolcott-Rallison syndrome is characterized by a severe beta-cell defect, and its pathogenesis includes PERK dysfunction [63]. In humans, a mutation the *WFS1 *gene encoding the ER transmembrane protein wolframin increases the incidence of DM in Wolfram syndrome patients [64].

A better understanding of ER stress in the pathogenesis of DM and its complications may provide novel research directions and therapeutic strategies. Some studies have shown that two chemical chaperones, phenyl butyric acid and tauroursodeoxycholic acid, can suppress ER stress in adipose tissue and liver tissue in obese mice, leading to a decrease in inflammatory signaling and increased insulin receptor signaling. This leads to enhanced circulating insulin sensitivity, normalizes blood glucose, and suppresses the progress of hepatic steatosis [65]. Research based on a lipid-induced ER stress model has shown that ER stress can inhibit apolipoprotein B 100 (apoB100) secretion in liver, which is the major factor promoting hepatic steatosis. Chemical chaperones can also suppress insulin resistance induced by inhibition of apoB100 secretion in liver [66]. The application of these compounds in the treatment of human diseases still has limitations because of the need for very high doses to be effective and because of undesirable pharmacokinetic effects. Further research is therefore needed to clarify how to enhance the ability of endogenous chaperones, promote protein folding, and enhance adaption of the UPR under metabolic stress in active metabolic tissue. This will help to develop new treatment strategies.

Another direction for therapeutic strategies is to reduce ER stress or regulate ER function, including directly targeting mediators of the UPR. Salubrinal is a small molecule that can inhibit eIF2*α* phosphorylation [67] and suppress cell death induced by ER stress in vivo and in vitro. Recently, a study has shown that salubrinal may activate the IRE1 pathway without causing JNK activation and may have a protective effect on islet cells [68]. Interestingly, some compounds that are currently in clinical trials or that have been used in T2DM treatment, such as PPAR agonists or salicylates, can affect the activity of key ER molecules [69, 70]. One research “hotspot” focuses on whether the therapeutic effects of these compounds are at least partly due to regulation of ER function or of the UPR. 

## 7. Conclusions

In recent decades, many studies have investigated the molecular mechanisms underlying the pathogenesis and progression of DM and complications associated with DM, with the aim of developing novel therapeutic strategies. Yet, the incidence of DM and its complications is still increasing worldwide, and need for development of new therapies targeting is therefore urgent. DM is therefore urgent. As stated above, the ER may be an intersection of integrated multiple stress responses and may be closely related to autophagy, apoptosis, and inflammation. The ER is therefore an attractive potential therapeutic target, and maintaining or improving ER function appropriately may prevent chronic metabolic disease. However, further studies to understand the theoretical and experimental basis of such a potential “organelle treatment” are required before such therapies can be applied.
